# Artificial Intelligence Enabled Lung Sound Auscultation in the Early Diagnosis and Subtyping of Interstitial Lung Disease

**DOI:** 10.3390/jcm14238500

**Published:** 2025-11-30

**Authors:** Avneet Kaur, Swathi Priya Cherukuri, Megha Shashidhar Handral, Hanisha Reddy Kukunoor, Rikesh KC, Swathi Godugu, Jieun Lee, Gayathri Yerrapragada, Poonguzhali Elangovan, Mohammed Naveed Shariff, Thangeswaran Natarajan, Jayarajasekaran Janarthanan, Jayavinamika Jayapradhaban Kala, Sancia Mary Jerold Wilson, Samuel Richard, Shiva Sankari Karrupiah, Dipankar Mitra, Vivek N. Iyer, Scott A. Helgeson, Shivaram P. Arunachalam

**Affiliations:** 1 Department of Internal Medicine, MedStar Union Memorial Hospital, Baltimore, MD 21218, USA; 2 Digital Engineering & Artificial Intelligence Laboratory (DEAL), Department of Critical Care Medicine, Mayo Clinic, Jacksonville, FL 32224, USA; swathicherukuri13@gmail.com (S.P.C.); drhanisha3391@gmail.com (H.R.K.); lee.jieun@mayo.edu (J.L.);; 3 Department of Computer Science & Computer Engineering, University of Wisconsin-La Crosse, La Crosse, WI 54601, USA; dmitra@uwlax.edu; 4 Division of Pulmonology, Department of Medicine, Mayo Clinic, Rochester, MN 55905, USA; 5 Division of Pulmonology & Department of Critical Care Medicine, Mayo Clinic, Jacksonville, FL 32224, USA

**Keywords:** interstitial lung disease (ILD), AI-based auscultation, lung crackles, early diagnosis, acoustic biomarkers, machine learning

## Abstract

**Background:** Interstitial lung disease (ILD) involves numerous chronic pulmonary conditions that damage the lung parenchyma and alveolar interstitium. ILD has overlapping clinical and radiological features with other commonly seen cardiac and respiratory conditions. If not identified and treated in a timely manner, it may lead to irreversible fibrosis and a poor prognosis in the patient. The current diagnostic methods are either invasive or reliant on imaging or specialist interpretation, which can lead to diagnostic delay, increased radiation exposure, and healthcare costs. Lung crackles, often under-recognized as a non-specific feature of ILD, may serve as an important diagnostic clue in identifying not only the early stages of ILD but also its subtypes. This review explores the potential of analyzing the lung sounds in ILD through AI-based auscultation. **Objective**: To provide a comprehensive analysis of the pathophysiological stages of lung injury in ILD, the specific acoustic features, and the location associated with each ILD subtype and to evaluate the current state-of-the-art non-AI and AI methodologies that are used to diagnose ILD. This review aims to analyze the limitations associated with the current modalities and to envision AI-integrated auscultation as a powerful, cost-effective, non-invasive, radiation-free screening tool for early detection of ILD and its subtypes. **Content Overview:** The review begins with a detailed analysis of the lung sound pathophysiology, exploring the two-stage mechanism of alveolar epithelial injury and fibrosis formation. Existing hypotheses explaining the mechanism behind crackle production and the role of structural anatomy and surface tension in the generation of pathological lung sounds are examined. A tabulated summary of common ILD subtypes is provided, including their inciting events, pathogenesis, anatomical auscultation locations, and prognostic implications. Current diagnostic modalities for ILD, both non-AI and AI-based, are summarized along with their limitations, emphasizing the need for improved diagnostic tools. **Discussion:** Existing studies suggest that AI-based auscultation can match or exceed the current modalities in its sensitivity and specificity for detecting ILD-related crackles. Clinicians can identify the specific sound pattern and then correlate it with the ILD subtype and understand the prognosis in real time, thereby providing timely intervention to the patient. Additionally, AI-based auscultation can be used in resource-limited settings and can potentially reduce dependence on pulmonology expertise and radiation-based imaging for monitoring the condition. **Conclusions:** This literature review highlights the clinical potential of AI-based auscultation for early and accurate diagnoses of ILD. Understanding the associated pathological sounds, biomarkers, and genetic mutations linked to different subtypes opens avenues for future development of non-invasive diagnostic panels for ILD in clinical practice.

## 1. Introduction

Interstitial lung disease (ILD) comprises several chronic pulmonary conditions that involve the lung parenchyma and alveolar interstitium [1]. According to the Global Burden of Disease Study 2019, the global incidence, mortality, and disability-adjusted life years (DALYs) related to ILD have increased by 118.6%, 166.63%, and 122.87%, respectively, from 1990 to 2019 [2]. This rising prevalence and mortality can be largely attributed to delays in diagnosis.

One major factor contributing to this delay is the nonspecific nature of ILD symptoms, such as dyspnea on exertion and chronic cough, which frequently overlap with common respiratory and cardiac conditions like chronic obstructive pulmonary disease (COPD), asthma, and heart failure [3]. Additionally, early structural and functional changes in ILD often go undetected on imaging and pulmonary function tests (PFTs), further complicating early diagnosis. As a result, by the time an accurate diagnosis is made, the disease has frequently progressed to irreversible fibrosis, which worsens long-term outcomes [4]. Studies have shown that with each year of diagnostic delay, there is an estimated 1.8% increase in the extent of fibrosis visible on chest CT scans [5].

### 1.1. Evolution of ILD Diagnosis

The diagnosis of ILD has undergone a significant transformation over the past few decades. Key modalities that have improved diagnostic accuracy include high-resolution computed tomography (HRCT), which provides detailed visualization of lung parenchymal involvement; pulmonary function tests (PFTs) such as DLCO and FVC, which assess disease severity; and, in select cases, lung biopsies for histopathological confirmation.

More recently, artificial intelligence (AI) has been integrated into these diagnostic modalities, further enhancing the diagnostic process. Clinicians can now use AI tools such as natural language processing (NLP) to identify specific fibrotic patterns on HRCT, detect abnormalities in PFTs, and analyze ILD-related terminology in clinical notes.

Despite their benefits, current diagnostic tools have notable limitations. PFTs are effort-dependent and thus susceptible to variability. NLP tools rely heavily on the quality of clinical documentation, which can lead to missed diagnostic cues if the notes are incomplete or vague. HRCT, the current gold standard, is resource-intensive, requires expert interpretation, and poses concerns related to radiation exposure and cost, making it unsuitable for routine screening. Furthermore, none of these modalities provides real-time, point-of-care diagnostics, which is essential for early detection and large-scale screening. These limitations highlight the need for a diagnostic approach that is accessible, non-invasive, radiation-free, and capable of detecting early physiologic changes before structural abnormalities appear. Prior studies have demonstrated that early ILD induces subtle biomechanical alterations that generate fine inspiratory crackles, and that AI-enabled acoustic analysis can detect these signals with high sensitivity even when HRCT and PFT results remain normal. This evidence provides a rationale for exploring AI-augmented auscultation as a complementary, frontline tool for early ILD detection.

### 1.2. Auscultation in ILD: A Missed Opportunity

Auscultation has long been a cornerstone of respiratory examination. However, with increasing reliance on imaging and other advanced modalities, its diagnostic potential in ILD has often been under-recognized. Auscultation can detect changes in airflow dynamics, transitions from laminar to turbulent flow, airway opening and closure, and fibrosis-related acoustic abnormalities.

Notably, early stages of ILD induce biomechanical changes in the lungs that can manifest as unique acoustic signals—sometimes even before they become evident on imaging. One such acoustic finding is fine inspiratory crackles, a hallmark of idiopathic pulmonary fibrosis (IPF), a major ILD subtype with a median survival of just three years. These subtle crackles are often overlooked by non-specialist physicians, delaying the initiation of antifibrotic therapy at stages where it would be most beneficial. However, traditional auscultation remains subjective and heavily dependent on the clinician’s skill, limiting its use as a reliable large-scale screening tool.

### 1.3. Bridging to AI: A Promising Solution

Artificial intelligence offers a promising solution to the challenges posed by traditional auscultation. Already revolutionizing fields such as ECG interpretation [6], echocardiography [7], imaging [8], and pathology [9], AI holds similar potential in redefining lung sound analysis.

AI-powered digital stethoscopes can detect lung sounds that are beyond the auditory threshold of the human ear. These tools use pattern recognition techniques to decode subtle acoustic changes—such as fine inspiratory crackles—associated with early fibrotic changes [10]. By identifying these signals earlier than imaging or PFTs can, AI-enhanced auscultation greatly improves diagnostic precision [11].

Moreover, early ILD involves biomechanical alterations such as surfactant dysfunction and micro-airway collapse, which also influence lung acoustics. Decoding these changes through AI models may allow for earlier detection and intervention. In addition to detecting ILD early, AI-based auscultation has the potential to differentiate and phenotype subtypes such as IPF, NSIP, hypersensitivity pneumonitis (HP), connective tissue disease-associated ILD (CTD-ILD), and rheumatoid arthritis-associated ILD (RA-ILD) [12].

Importantly, AI-based tools may also uncover genetic insights by identifying molecular-level surfactant dysfunctions linked to mutations such as *SFTPC*, *ABCA3*, and *TERT*, based on the acoustic signatures they produce. Because AI-based auscultation is non-invasive, widely accessible, and does not rely heavily on specialist interpretation, it holds the potential to become a powerful point-of-care tool. By integrating phenotypic and genomic information, it may eventually serve as an acoustic fingerprint of ILD subtypes, supporting personalized treatment strategies and advancing precision medicine [10].

Compared with previous work that has largely treated lung sound analysis, imaging, and biomarker research as separate domains, this review offers an integrative, pathophysiology-anchored framework for AI-enabled auscultation in ILD. It connects the biomechanics of alveolar injury and fibrosis with subtype-specific acoustic signatures, maps these signatures to anatomical auscultation locations, and aligns them with contemporary AI architectures for lung sound and imaging analysis. In addition, this review emphasizes the potential of AI-enhanced auscultation as a frontline, radiation-free screening tool that can be deployed in primary care and resource-limited settings, rather than functioning solely as an adjunct in tertiary centers. By synthesizing mechanistic insights, clinical auscultation patterns, and multimodal AI strategies across lung sounds, HRCT, and biomarkers, this work outlines a translational roadmap for the use of AI-integrated auscultation in early diagnosis, ILD subtype stratification, and precision-guided disease management.

Despite major advancements, the mechanisms underlying lung sound generation in ILD—and the diagnostic potential of AI-based auscultation—remain underexplored. This review aims to examine the physiological basis of lung sound production in ILD, the limitations of current diagnostic approaches, and the emerging role of AI-powered auscultation in capturing disease-specific acoustic signals.

The central proposition of this review is that AI-based auscultation can transform future pulmonary practice by providing a real-time, bedside diagnostic tool for early ILD detection and by complementing existing modalities within multimodal diagnostic frameworks. Furthermore, it may help monitor disease progression and treatment response, reduce radiation exposure, and minimize the need for repeated imaging. In an era where diagnostic delay can lead to irreversible damage and poor outcomes, reimagining auscultation through the lens of AI can be a significant transformation in ILD care.

## 2. Methods

### 2.1. Study Design and Ethics

This work is a narrative, non-systematic review that synthesizes evidence on ILD pathophysiology, lung sound generation, and AI-enabled diagnostic tools, with a focus on AI-augmented auscultation. No new human or animal data were collected; therefore, institutional review board approval, informed consent, and an ethics reference number were not required.

### 2.2. Literature Search

A focused literature search was performed in PubMed/MEDLINE, Embase, Web of Science, IEEE Xplore, and Google Scholar. The main search was conducted between January 2024 and April 2025, with no lower year limit to allow inclusion of foundational ILD and lung sound studies. Search terms combined concepts related to the following:

Disease domain: “interstitial lung disease”, “idiopathic pulmonary fibrosis”, “progressive fibrosing ILD”, “connective tissue disease-associated ILD”, “hypersensitivity pneumonitis”;

Auscultation domain: “lung sounds”, “crackles”, “Velcro crackles”, “phonopulmography”, “digital stethoscope”;

AI/quantitative domain: “artificial intelligence”, “machine learning”, “deep learning”, “convolutional neural network”, “MFCC”, “STFT”, “wavelet”, “quantitative CT”, “radiomics”, “biomarkers”.

Reference lists of key ILD, auscultation, and pulmonary-AI articles and guidelines were also hand-searched to identify additional relevant studies.

Only English-language, full-text articles were considered.

### 2.3. Eligibility and Data Synthesis

The review included the following: clinical and pathophysiological ILD studies; work on lung sounds and crackle mechanisms; AI/signal-processing studies on respiratory acoustics; AI applications in ILD imaging, PFTs, and biomarkers; implementation, fairness, and regulatory papers relevant to pulmonary AI.

Conference abstracts without sufficient methodological detail, animal-only studies without clear translational relevance, and non-scholarly opinion pieces were excluded.

Because of the heterogeneity of designs, datasets, and AI architectures, no formal statistical pooling or meta-analysis was performed. Instead, evidence was synthesized narratively in thematic sections (pathophysiology and lung sounds, AI for auscultation, AI for imaging and functional tests, and multimodal integration), emphasizing diagnostic performance, clinical applicability, and gaps for future research.

## 3. Pathophysiology of Lung Sound Production in ILD

Interstitial lung disease (ILD) is a diverse group of lung disorders that involves inflammation and fibrosis of the pulmonary interstitium, leading to poor gas exchange and a progressive decline in lung function. The hallmark progression of ILD typically follows two stages:

Injury to the alveolar epithelium, which then triggers an abnormal repair response

Fibroblast proliferation and deposition of extracellular matrix (ECM), leading to fibrosis

These stages are summarized below:

Stage 1: Alveolar epithelial injury and inflammation

Often multifactorial or idiopathic, common triggers that result in injury to type II alveolar epithelial cells (AEC II) include cigarette smoke, environmental exposure, autoimmune conditions, genetic predisposition, and certain medications. AEC II cells primarily produce surfactants that are responsible for repairing the alveolar surface. Repetitive injury to the alveolar epithelium can lead to abnormal wound healing and dysregulated epithelial–mesenchymal transition (EMT), in which epithelial cells acquire mesenchymal properties, leading to the accumulation of fibroblasts and myofibroblasts [13].

In the early stages of the disease, this injury is characterized by interstitial edema and inflammation, which results in thickening of the alveolar-capillary membrane and impaired gas exchange. The inflammation then lays the foundation for further fibrosis and lung remodeling.

Stage 2: ECM deposition and irreversible lung injury

The healing process by the AECs becomes excessive, and transforming growth factor-beta (TGF-β) activates the accumulated fibroblasts into myofibroblasts, which produce collagen and ECM proteins [14]. Progressive ECM deposition disrupts normal alveolar architecture, reduces lung compliance, and contributes to a restrictive lung pathology. As fibrosis advances, the lung tissue becomes increasingly thick and stiff, further impairing its ability to expand and ultimately leading to irreversible lung damage.

### 3.1. Mechanism of Lung Sound Production in ILD

The mechanical changes in lung architecture, including fibrosis and airway distortion, lead to the production of pathological lung sounds—particularly crackles. Over the years, various hypotheses have been proposed to explain the generation of these crackles. The most widely accepted is the Stress-Relaxation Quadrupole Hypothesis [15].

Due to fibrosis and scarring of the lung tissue in ILD, the airways become stiff, leading to the collapse of distal airways during expiration. This results in gas trapping and surface tension buildup, referred to as “stress.” During inspiration, as the patient inhales, the increasing intrathoracic pressure causes abrupt reopening of these collapsed distal airways and alveoli—a process termed “relaxation.” This sudden opening produces the characteristic “pulling-apart” sound, often described as Velcro-like crackles. These sounds are most prominent in gravity-dependent areas of the lungs, such as the bilateral basilar zones. The sound waves spread out in a unique four-way pattern during airway reopening—a phenomenon known as a “quadrupole” [15].

Another hypothesis explaining the predominance of crackles during inspiration is the Polarity Theory by Vyshedskiy et al. According to this theory, as inspiratory pressure builds, the airways suddenly pop open, and lung tissue moves outward—away from the chest wall. This outward motion creates “negative polarity,” as the sound waves move away from the microphone of the stethoscope. This sudden, forceful release of pressure is a high-energy event, generating loud, sharp crackles. In contrast, during expiration, the airways collapse gradually, the lung tissue moves inward, and “positive polarity” is produced. However, due to the slower and less forceful nature of collapse, expiration generates softer and less frequent crackles [16]. In addition to these primary hypotheses, several other mechanisms have been proposed to explain crackle generation:

Airway Reopening Due to Surface Tension Dynamics: In ILD, damage to AEC II cells causes surfactant dysfunction, increasing surface tension and causing alveolar collapse. Higher inspiratory pressures are required to overcome this tension, leading to sudden reopening and crackle production [17]. Trapped Gas Hypothesis: This suggests that air becomes trapped in distal lung regions during expiration due to airway closure. Upon inspiration, increased airway pressure causes sudden reopening, resulting in crackles. However, this hypothesis is limited because crackles are also heard during expiration, not just inspiration [18]. Air Bubbling Through Secretion Hypothesis: In ILD, inflammation and edema can lead to secretions in the airways. As air moves through this fluid, bubbling sounds may be produced. This mechanism is more typical in pneumonia and bronchitis, but may contribute to crackles in ILD [19]. Mechanical Stress from Structural Changes: In advanced ILD, distortion of lung architecture, traction bronchiectasis, and honeycombing can cause abrupt airway opening and closure, leading to crackles. Some studies have noted that crackle pitch varies with lung volume. During inspiration, more small-diameter airways are recruited, increasing lung volume and raising crackle pitch during this phase of the respiratory cycle. Table 1 summarizes the major ILD categories, their representative subtypes, typical auscultation findings, and associated prognostic patterns. This table highlights how specific acoustic signatures—particularly fine inspiratory crackles—vary across ILD etiologies and correlate with disease severity.

**Figure 1:**
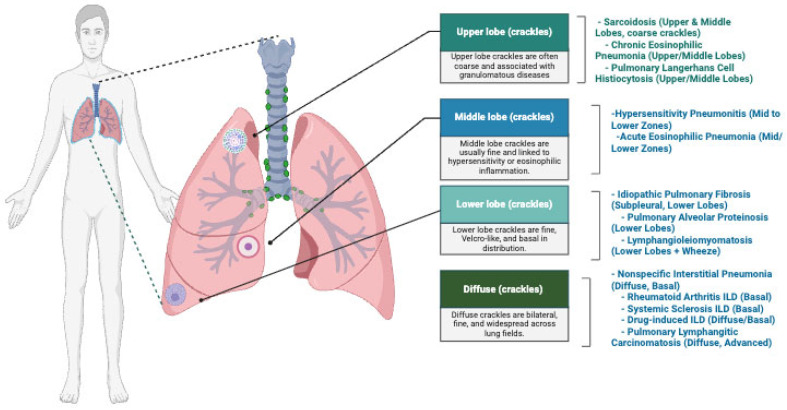
Prominent auscultation locations in subtypes of ILD.

Though crackles are mostly heard in the lower parts of the lungs, different ILD subtypes exhibit crackles in specific regions of the lungs depending on the pattern of fibrosis. In idiopathic pulmonary fibrosis (IPF), fibrosis is more pronounced in the subpleural regions and lower lobes, producing characteristic ‘Velcro-like’ crackles in the later stages of the disease. On the other hand, nonspecific interstitial pneumonia (NSIP) also affects the lower lobes but in a uniform and diffuse pattern. Due to its less aggressive nature, the crackles are milder compared to those in IPF. Another subtype, hypersensitivity pneumonitis (HP), triggered by inhaled environmental antigens, causes inflammation of the bronchioles and mid-lung zones, producing crackles in these areas. Granulomatous conditions such as sarcoidosis, which have multisystem involvement, can produce granulomas in the upper lobes, leading to coarse crackles in those regions. In less common subtypes like lymphangioleiomyomatosis (LAM), airway narrowing and cystic changes produce lower lobe crackles, often accompanied by wheezing. A similar pattern of crackles is heard in pulmonary alveolar proteinosis (PAP), which affects both lower lobes due to proteinaceous material deposition in the alveoli. Figure 1 illustrates the anatomical distribution of crackles across upper, middle, lower, and diffuse lung zones and links each auscultation region with ILD subtypes typically associated with those acoustic patterns. This mapping demonstrates how specific crackle locations correspond to underlying disease mechanisms, including granulomatous disorders (upper lobe), hypersensitivity or eosinophilic inflammation (middle lobe), fibrotic diseases such as IPF (lower lobes), and basal/diffuse involvement seen in autoimmune and advanced ILDs.

Understanding the underlying fibrosis pattern in various ILD subtypes and the regional variation in auscultatory findings is essential to differentiate between them early, aiding prompt diagnosis and management.

### 3.2. Clinical Implications of the Auscultatory Findings

The traditional auscultation techniques used by clinicians have certain challenges in ILD, particularly in subclinical, silent, or early stages of ILD, where the crackles are often subtle and can be difficult to hear in clinical settings. However, with the advancements of digital stethoscopes, which can amplify the lung sounds and AI tools that can analyse the frequency, timing, and pattern of the sound production, auscultation has the potential to become a powerful diagnostic tool for ILD. Understanding the pathophysiological mechanisms of sound production in ILD can tremendously help clinicians in identifying the potential areas to auscultate and even differentiate between the subtypes of ILD based on the location and the characteristics of sound produced, thus enabling early detection and timely intervention of the disease.

## 4. AI Techniques in ILD: Detection and Processing of Lung Sounds and Imaging

Artificial Intelligence (AI) is reshaping how interstitial lung diseases (ILDs) are detected, tracked, and managed—especially in early stages where traditional clinical assessments may fall short. ILD diagnosis has long depended on pulmonary auscultation and high-resolution computed tomography (HRCT); however, both methods are subject to inter-observer variability and a lack of quantitative reproducibility. AI now offers powerful tools to augment these modalities through signal processing, feature extraction, and machine learning.

### 4.1. AI in Lung Sound Analysis

In auscultation, AI enables scalable and objective interpretation of respiratory sounds, helping detect early fibrosis by identifying fine inspiratory “Velcro” crackles, which are often missed in conventional clinical practice due to subjectivity and human auditory limitations [52]. AI-driven auscultation begins with the acquisition of raw lung sounds using electronic stethoscopes, typically stored in WAV format. These audio signals undergo preprocessing using advanced signal transformation techniques like:

Mel-Frequency Cepstral Coefficients (MFCCs)—replicate human auditory perception [53]

Short-Time Fourier Transform (STFT)—captures time-varying frequency content [52,53]

Wavelet Transforms—ideal for highlighting transient signals such as crackles [52]

The extracted features are then fed into deep learning models for classification or analysis of disease progression. Depthwise Separable Convolutional Neural Networks (DS-CNNs) have emerged as a lightweight and real-time solution for mobile or primary care settings, achieving 85.74% accuracy in Velcro crackle detection using fused MFCC and STFT inputs [53]. Traditional deep architectures, such as VGG-16 and ResNet, offer higher representational capacity but are computationally intensive [52].

A key innovation in this domain is the Bi-ResNet (Bidirectional Residual Network), which allows bidirectional information flow during feature propagation, significantly improving crackle classification with 97.82% accuracy, while maintaining a lower parameter count than ResNet-101 or DenseNet—ideal for embedded systems [54].

To further model the temporal dynamics of breath sounds, CNN–LSTM hybrid architectures have been proposed, combining spatial feature learning with time-series modelling across respiratory cycles [52]. Additionally, Transformer-based models, though still in early development, offer promise in learning long-range temporal dependencies for tracking evolving acoustic changes in ILD [52].

Ensemble learning techniques have also shown promise for telemedicine-based respiratory diagnostics. A framework developed by Jaber et al. employed MFCCs combined with Daubechies wavelet transform (db4) features and applied multiple classifiers, including an improved Random Forest, AdaBoost, and Gradient Boosting, to classify seven types of lung sounds (e.g., fine/coarse crackles, wheezes, stridor, squawk). The improved Random Forest classifier achieved the highest accuracy (99.04%), demonstrating strong potential for remote ILD detection in resource-limited or home-based settings [55].

These approaches not only aid in early fibrosis detection but also provide a scalable framework for remote monitoring and telemedicine-based ILD screening. A comparative summary of these AI architectures and their performance metrics is provided in Table 2.

### 4.2. AI Beyond Lung Sounds: Toward Multimodal Early ILD Detection

While lung sound-based AI models offer scalable and non-invasive tools for early ILD detection, combining them with additional clinical data streams can significantly improve diagnostic accuracy. AI models trained on electronic health record (EHR) data—such as age, gender, smoking status, comorbidities, and medications—can identify risk profiles and refine pretest probability [55]. Similarly, laboratory data, including blood biomarkers such as KL-6 and SP-D [56]. Inflammatory markers like C-reactive protein (CRP) [57] have been integrated into deep learning models for predicting ILD exacerbation and mortality [57], and provide insights into epithelial damage, inflammatory activity, and disease severity. Pulmonary Function Tests (PFTs), such as FVC and DLCO, are functional correlates of ILD severity and have been successfully incorporated into machine learning-based diagnostic frameworks, improving clinician performance when used with explainable AI support [58].

In parallel, HRCT imaging-based AI, particularly using context-sensitive SVM (csSVM) and emerging 3D convolutional and attention-based architectures, enhances ILD subtype classification—especially when the disease remains subclinical [59]. Studies combining auscultation, imaging, EHR, and lab data report higher sensitivity and specificity for early-stage ILD diagnosis, supporting real-world deployment in telemedicine and primary care settings.

**Figure 2:**
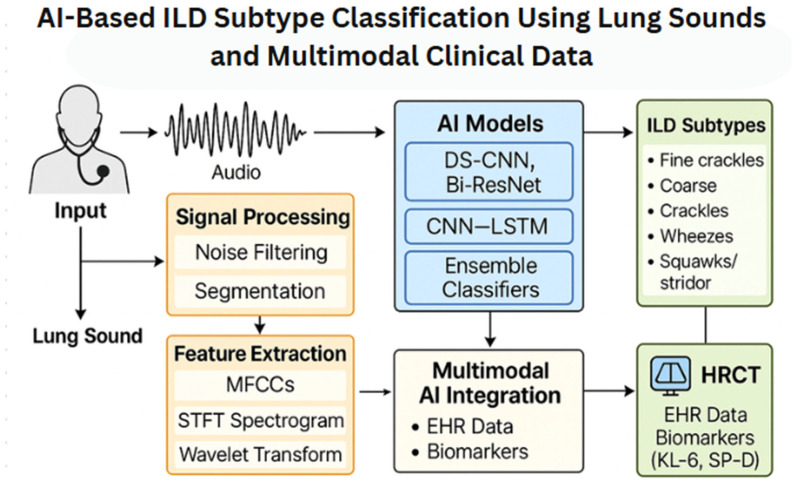
Multimodal Workflow for AI-Based ILD Subtype Classification.

Thus, future AI-driven diagnostic frameworks should aim for multimodal integration, enabling more holistic and personalized ILD screening illustrated in Figure 2. This diagram shows how different types of data are combined to help detect interstitial lung disease (ILD) subtypes more accurately. It starts with lung sounds recorded through a digital stethoscope. These sounds are cleaned (noise filtering), broken down into meaningful segments, and then analyzed using features like MFCCs, spectrograms, and wavelet transforms. These features are passed through advanced AI models—including DS-CNN, Bi-ResNet, and CNN–LSTM—to identify abnormal sound patterns. At the same time, clinical information like patient records and biomarkers (e.g., KL-6 and SP-D) is added to improve accuracy. HRCT scans and clinical features help confirm the final diagnosis of different ILD subtypes, such as fine crackles, coarse crackles, wheezes, and squawks/stridors. Figure 2 provides an overview of the AI-based workflow for ILD subtype classification using lung sounds and multimodal clinical data. The diagram outlines how raw auscultatory audio undergoes noise filtering and segmentation, followed by feature extraction using MFCCs, STFT spectrograms, and wavelet transforms. These features are then processed through contemporary AI architectures—including DS-CNN, Bi-ResNet, CNN–LSTM, and ensemble models—to identify ILD-related acoustic phenotypes. The workflow also illustrates how lung sound-derived features can be integrated with HRCT findings, biomarkers (e.g., KL-6, SP-D), and EHR data to support multimodal ILD subtype classification.

## 5. Current Diagnostic Modalities for ILD

Over the years, researchers and clinicians have explored a wide variety of methods to better understand and manage interstitial lung diseases (ILDs). These approaches range from traditional tools—such as pulmonary function tests (PFTs), high-resolution CT scans (HRCT), six-minute walk tests (6MWT), and blood-based biomarkers—to more recent advances involving artificial intelligence (AI). While non-AI methods remain central to routine clinical care, AI-based techniques are quickly gaining ground for their ability to analyze complex data, automate image interpretation, and support early diagnosis and risk prediction. Both categories of methods offer unique strengths and serve different clinical needs. To provide a clear overview, these diagnostic and prognostic modalities—along with their techniques, findings, and limitations—are summarized in Table 3 below.

## 6. Discussion

### 6.1. AI in General Medicine

Artificial intelligence, since its emergence, has transformed traditional diagnostic and therapeutic approaches in clinical practice. Over the years, AI has evolved through deep learning (DL) and machine learning (ML) models, which have the ability to analyze large datasets using pattern recognition and high-speed processing. AI has emerged as a powerful tool with applications across multiple domains of medicine, including diagnostic imaging, pathology, disease monitoring, and many other areas [100].

Role of AI in Pulmonology

In the field of pulmonology, AI has been integrated into imaging analysis, such as HRCT, and used for the clinical assessment of chronic pulmonary conditions, including asthma, emphysema, and COPD [101]. AI prediction models also serve as screening tools for sleep apnea [102] and have been employed in ventilator management in intensive care settings [103].

Despite these advancements, some subdomains of respiratory medicine—such as interstitial lung disease (ILD)—have not been appropriately emphasized.

### 6.2. Challenges in ILD Diagnosis

ILD includes numerous subtypes, each with varying degrees of inflammation and fibrotic lung damage. These subtypes differ significantly in management and prognosis, making early and accurate diagnosis crucial. One of the biggest challenges in diagnosing ILD is the overlap in clinical features with other common respiratory and cardiac conditions, such as asthma, COPD, and congestive heart failure, which are frequently encountered by clinicians in routine practice.

This overlap often leads to missed recognition of early disease, especially in non-specialist settings. Studies have documented a median diagnostic delay ranging from 7 months to 2 years from the onset of symptoms to the final diagnosis in ILD patients [104]. During this delay, patients often receive incorrect or suboptimal treatment, resulting in further deterioration of lung function [105].

Prompt initiation of appropriate therapy, especially in progressive fibrosing phenotypes, is essential and can significantly improve clinical outcomes [106].

### 6.3. Limitations of the Current Diagnostic Paradigms

It is essential to critically evaluate the current ILD diagnostic framework and identify the limitations and obstacles in the early diagnosis of the condition. Traditional diagnostic tools for ILD include clinical examination, auscultation, imaging, pulmonary function tests (PFTs), and, in some cases, invasive procedures [107]. While this approach is comprehensive, there are key limitations at each step that contribute to delayed or missed diagnoses.

High-resolution CT (HRCT) of the chest, the gold standard for identifying interstitial abnormalities, can detect reticular patterns, ground-glass opacities, and honeycombing to assess the degree of fibrosis and distinguish between ILD subtypes [108]. However, its high cost and radiation exposure make it unsuitable for routine use in resource-limited or rural settings.

With growing demand for CT imaging, studies have shown a global annual increase in chest CT scans, which places significant pressure on radiologists, contributing to underreporting and diagnostic delays.

Moreover, PFT parameters such as forced vital capacity (FVC) and diffusing capacity for carbon monoxide (DLCO), though commonly used for disease monitoring, can be normal in the early stages of non-fibrotic ILD or in patients with comorbidities that might obscure the clinician’s interpretation [109].

In cases where imaging is inconclusive, clinicians may pursue invasive techniques like bronchoscopy with transbronchial biopsy or surgical lung biopsy. However, the risks of these procedures, especially in patients with poor pulmonary reserve or advanced disease, often make them infeasible. The need for specialized personnel further limits access and contributes to diagnostic delays, particularly in community-based healthcare systems.

### 6.4. Auscultation: An Underrecognized Tool in ILD Diagnosis

Auscultation, one of the most widely used non-invasive clinical tools, has long been part of routine respiratory examinations. Yet, its role in diagnosing ILD remains underrecognized [110]. Traditional auscultation relies on the physician’s auditory ability, clinical expertise, and the quality of the stethoscope. In early ILD, findings such as fine crackles can be too subtle to detect and are often missed, especially in routine or non-specialist settings, due to poor interobserver agreement [111].

Additionally, there is no standardized technique used across clinicians to record or interpret lung sounds. Unlike imaging or lab investigations—which are stored in the patient’s medical record—auscultatory findings are rarely recorded or retained, limiting their value for longitudinal assessment and clinical follow-up.

### 6.5. AI in Established ILD Modalities

In recent years, AI has increasingly been integrated into established diagnostic tools for ILD, assisting in clinical decision-making. However, despite these advancements, most tools fail to identify patients in early stages, when timely intervention could have the greatest impact on long-term prognosis.

Advanced AI algorithms are now used to interpret HRCT scans, quantifying fibrosis and recognizing subtype-specific imaging patterns. While these tools enhance diagnostic confidence, they are largely confined to tertiary-care settings [112].

Similarly, ML algorithms have been applied to PFT interpretation, allowing clinicians to detect subtle or atypical patterns in spirometry and to stratify disease risk. However, like HRCT, these tools are typically used only after ILD is already suspected, and an initial diagnostic workup has been initiated [79].

AI has also been deployed in electronic health records (EHRs) through risk-scoring systems that analyze data such as demographics, comorbidities, medication use, and clinical notes to identify high-risk patients. However, these systems are highly dependent on the quality of documentation and its specificity to ILD—which is often lacking, especially in primary care or community-based settings [113].

These modalities do not address the critical frontline gap in ILD diagnosis. There remains a need for a widely accessible, low-cost screening tool that can be used in primary care or rural environments. This unmet need creates a unique opportunity to reimagine the stethoscope itself through the transformative lens of AI.

### 6.6. The Potential of AI-Enhanced Auscultation

With the advent of digital stethoscopes in the late 20th century, the recording and transmission of lung sounds have become more precise and scalable. The integration of digital signal processing and AI techniques has further revolutionized auscultation.

Today, pathological lung sounds can be analyzed using techniques like the Fourier transform and wavelet analysis. Additionally, deep learning (DL) models, especially convolutional neural networks (CNNs), have demonstrated the ability to detect complex respiratory patterns, including inspiratory crackles in early-stage ILD, with high accuracy [84].

Emerging technologies such as dual-mode sensors, wearable devices, and explainable AI (XAI) are further expanding the diagnostic landscape.

Using ML or DL algorithms to process lung sounds recorded via digital sensors, clinicians can identify early fibrotic changes linked to fine inspiratory crackles [114]. Research has shown that these crackles may appear before structural changes are visible on HRCT or functional impairment is detected on PFTs.

### 6.7. Real-World Deployment and Clinical Trial Evidence

There is a growing body of evidence supporting the real-world effectiveness of AI-based auscultation tools. For example, a study by Aykanat et al. demonstrated that convolutional neural networks trained on lung auscultation recordings achieved high diagnostic accuracy for respiratory diseases, comparable to expert assessment. These results highlight the effectiveness of AI models in differentiating comorbid pulmonary conditions [115].

However, limitations remain, particularly in terms of longitudinal validation. More evidence is needed regarding AI auscultation’s impact on time to diagnosis, initiation of appropriate therapy, and long-term quality of life.

Taken together, the evidence across clinical trials, telemedicine deployments, and HRCT-AI studies suggests that AI-augmented auscultation offers complementary diagnostic value rather than duplicating existing modalities. Prior studies have shown that early inspiratory crackles precede radiological abnormalities and correlate with early fibroproliferative changes, supporting the concept that acoustic biomarkers may capture pathophysiology at a stage when HRCT and PFTs remain normal. These findings align with earlier work by Kim et al. and Aykanat et al., who demonstrated high sensitivity of DL-based crackle detection even in early disease. This convergence across studies reinforces the potential of AI-auscultation to narrow the diagnostic delay documented in ILD literature and to serve as a frontline screening tool, particularly in primary care and resource-limited settings.

### 6.8. Advantages of AI-Based Auscultation

AI-based auscultation offers the potential to become a non-invasive, cost-effective, and widely accessible tool for the early detection and monitoring of ILD, especially in settings where conventional imaging is unavailable or unaffordable.

By expanding access to this technology in underserved regions, AI-powered stethoscopes can help identify a hidden burden of ILD that likely exceeds current estimates. Training primary care providers and nurses in the use of AI stethoscopes can bridge the gap between symptom onset and specialist referral, enabling timely triaging and early intervention [116].

This approach may also reduce unnecessary imaging and invasive procedures, thereby lessening the burden on specialists. As these tools require only a digital stethoscope and a connected device, they are easy to incorporate into telemedicine workflows, allowing experts to remotely assess lung sounds [117].

Moreover, their portability and radiation-free nature make them ideal for longitudinal follow-up in resource-limited settings. AI algorithms can also reduce interobserver variability, a key limitation of traditional auscultation, by offering standardized, consistent interpretations and the ability to save recordings for future review [118].

### 6.9. Comparative Advantages over Traditional and Emerging Diagnostic Modalities

AI-based auscultation has multiple advantages over pulmonary function testing and HRCT—particularly its capability to detect ILD earlier and be deployed at the primary care level. It is affordable, non-invasive, and more portable than HRCT, which is costly, involves radiation exposure, and requires specialized infrastructure and personnel. HRCT also requires day-of coordination, often limiting access in remote or underserved areas.

While PFTs provide valuable information on lung function, their sensitivity is reduced in early-stage ILD or when subtle parenchymal abnormalities are present. They are also effort-dependent, which can compromise accuracy in certain patient populations. In contrast, AI-based auscultation can detect minute changes in lung acoustics, thanks to ML models trained on large, diverse datasets [119].

Furthermore, unlike AI-assisted ECGs or AI-supported fundoscopy—tools increasingly adopted in other specialties—AI-auscultation offers real-time, bedside monitoring and is especially useful in pulmonary medicine for ongoing, non-invasive surveillance. Unlike bronchoscopy, it does not require high-level training or procedural equipment [120].

As it is housed in handheld digital stethoscopes, AI-auscultation supports time-efficient diagnosis and clinical decision-making in primary care, potentially addressing healthcare shortages in low-resource regions [10].

### 6.10. Clinical Effectiveness: Evidence from Trials and Real-World Validation

Recent clinical trials have demonstrated the high efficiency of AI-based auscultation tools in detecting pathological lung sounds, including subtle crackles and wheezes, which are critical for diagnosing early stages of ILD.

For instance, Kim et al. (2021) showed that deep learning algorithms achieved over 90% sensitivity and specificity in identifying and classifying adventitious lung sounds, thereby enhancing pulmonologist assessments [121]. These findings have been validated in real-world applications.

Mayo Clinic, for example, has incorporated AI-enhanced auscultation into telemedicine workflows, significantly improving the early identification of ILD in remote settings and supporting more effective patient triaging [122]. Similarly, Butterfly Network’s handheld ultrasound and digital stethoscope platforms utilize AI to guide lung examinations, aiding clinicians in both hospital and primary care environments [123].

Despite these promising developments, further large-scale randomized and pragmatic trials are needed to assess clinical outcomes, cost-effectiveness, integration feasibility, and interoperability across healthcare systems.

### 6.11. Addressing Global Equity and Accessibility Gaps

Interstitial lung diseases often go undiagnosed in remote and underserved areas due to limited access to HRCT, PFTs, and pulmonologists, resulting in poor prognoses [124]. Phonopulmography, when integrated with AI, can analyze acoustic patterns to detect fine crackles—an early marker of fibrotic ILD—and help identify high-risk subtypes such as hypersensitivity pneumonitis and systemic sclerosis-associated ILD with high sensitivity [125].

These tools are well-suited for use by frontline healthcare workers, enabling deployment in areas without advanced infrastructure. Early identification and triaging using AI-enhanced auscultation could substantially reduce morbidity and mortality associated with ILD in low-resource settings [126].

### 6.12. Global Health Access and Deployment Strategies

One of the most important considerations for real-world adoption of AI-based tools is their potential to promote global equity. In many low- and middle-income countries, access to HRCT and spirometry is limited due to cost constraints, inadequate infrastructure, and workforce shortages [127].

Mobile-based auscultation tools, integrated with deep learning algorithms, can be embedded into primary care clinics or even community health kits, particularly where pulmonologists are scarce.

To ensure equitable deployment [128], however, successful integration will require the following:

1. Partnerships with local health authorities.
2. Language-agnostic algorithm development.
3. Robust community engagement strategies.

### 6.13. The Future of ILD Stratification Through Auscultatory Signatures

The integration of AI into auscultatory signal analysis offers a transformative pathway for stratifying and managing ILD subtypes. AI-enhanced phonopulmography can identify phenotype-specific acoustic patterns, potentially distinguishing between forms such as idiopathic pulmonary fibrosis (IPF), acute interstitial pneumonia (AIP), nonspecific interstitial pneumonia (NSIP), and hypersensitivity pneumonitis (HP).

In connective tissue disease-associated ILDs, such as systemic sclerosis-associated ILD and rheumatoid arthritis-associated ILD, subtle acoustic changes may be missed by clinicians but detected by machine learning algorithms.

Emerging studies suggest that Velcro-like crackles in IPF differ in timing, frequency, and distribution compared to those in NSIP or HP [129]. Even rare ILDs, such as sarcoidosis, lymphangioleiomyomatosis (LAM), and pulmonary alveolar proteinosis (PAP), may exhibit unique auscultatory signatures, offering a new opportunity for early, non-invasive subtype identification.

This acoustic subclassification can support precision-guided management, especially in subtypes where early treatment can significantly alter prognosis [130].

### 6.14. Integration with High-Risk Screening Protocols

AI-augmented phonopulmography has also shown promise in screening for high-risk pulmonary conditions, including COVID-19 and occupational lung diseases like silicosis. Its ability to detect very subtle inspiratory crackles enables early identification, often before symptoms become apparent or structural changes are visible [79].

Incorporating PPG into routine health check-ups or high-risk occupational screenings can reduce reliance on HRCT, which is impractical for repeated use due to radiation exposure, cost, and infrastructure needs [131]. AI also helps mitigate interobserver variability, ensuring consistent and objective follow-up—regardless of clinician or setting. When combined with PFTs or emerging biomarkers like liquid biopsy, PPG can form the foundation of a multimodal screening paradigm targeted at vulnerable populations [126].

### 6.15. Interoperability and Health System Integration

Integrating AI-auscultation into electronic health records (EHRs) and clinical decision support systems (CDSSs) is a critical milestone for ensuring its role in longitudinal disease monitoring. Interoperable systems can continuously capture, store, and analyze respiratory sound patterns, allowing clinicians to track disease progression and receive automated alerts when abnormalities are detected. Recent studies have demonstrated that AI algorithms can identify fine crackles, wheezes, and other abnormal sounds with high sensitivity. When linked to EHRs, these findings can support risk stratification, personalized treatment planning, and even remote consultations [132].

In resource-limited or remote settings, AI-based auscultation integrated into telemedicine or home-monitoring platforms can provide real-time insights, aligning with global efforts in healthcare digitization and serving as a backbone for predictive modeling and surveillance of chronic lung disease [133,134].

### 6.16. Innovations in Machine Learning for Pulmonary AI

Recent advancements in machine learning continue to accelerate progress in pulmonary AI, but significant challenges remain. One key limitation lies in supervised learning, which requires large volumes of labeled training data, often curated through expert annotation. This process is time-intensive and may not fully capture the clinical variability seen across diverse populations and healthcare settings [134].

Furthermore, supervised models tend to underperform when applied to new geographic regions or when using different data acquisition devices, due to biases in the original training datasets [135].

To overcome these challenges, researchers are now exploring federated learning and self-supervised learning as promising alternatives. Federated learning allows shared model training across institutions while preserving patient privacy, improving generalizability without requiring data to be centralized [136].

These methods offer the potential to enhance model robustness, improve applicability across global populations, and accommodate hardware variability, thereby enabling broader deployment of AI-auscultation systems.

## 7. Future Directions

Future studies should prioritize training AI models on large, technically diverse datasets to ensure greater generalizability across patient populations and clinical environments. Real-world validation studies comparing AI-auscultation with gold standard HRCT findings are essential for building clinical trust and regulatory confidence. Routine application of AI-auscultation could also help in monitoring disease progression, evaluating treatment response, and reducing the need for repeated imaging or invasive testing [137].

### 7.1. Multimodal Integration: The Future of AI in ILD Diagnosis

A major milestone in AI development will be the fusion of multiple diagnostic data streams. Combining auscultatory findings with data from HRCT, PFTs, genomics, and biomarkers can create a more comprehensive and cost-effective diagnostic approach for ILD [138].

HRCT provides detailed anatomical visualization.

PFTs assess functional decline.

Genetic and biomarker profiles support personalized therapy.

By integrating these modalities, clinicians can achieve early, precise, and efficient diagnosis, improving care for complex and heterogeneous ILD subtypes.

### 7.2. Fairness Metrics: Ensuring Equity in AI-Auscultation

A critical component in deploying AI for healthcare is ensuring fairness across demographic groups. AI models trained predominantly on data from specific regions or populations may fail to perform adequately in underrepresented communities [139].

To address this, researchers must incorporate fairness metrics, such as performance stratification by age, gender, ethnicity, socioeconomic status, and comorbidity burden. These metrics assess whether the model delivers consistent, accurate predictions across all subgroups. Bias mitigation must occur during both development and deployment. Ensuring inclusivity in training datasets enhances generalizability and ensures marginalized communities benefit equally—particularly important in ILD, where diagnostic delays are more common among underserved populations [140,141].

### 7.3. Policy Recommendations: National AI-Health Initiatives

Widespread adoption of AI-auscultation will require strong national support and regulatory frameworks. Countries such as the UK, India, and the USA have already launched major initiatives to facilitate the ethical integration of AI in healthcare.

The UK NHS AI Lab focuses on validating and safely deploying AI tools [142].

India’s National Digital Health Mission is expanding digital health infrastructure to support AI-based care.

The US FDA has established regulatory pathways for AI-driven medical devices to balance innovation and safety.

These national efforts offer models for international collaboration, clinical translation, and global standardization of AI-based tools.

### 7.4. Patient Trust and Transparency: Building Confidence

Patient acceptance is vital for successful implementation. Trust increases when AI tools are clinically accurate, transparent, and easy to understand [143].

One solution is Explainable AI (XAI), which clarifies how decisions are made based on lung sound features. This empowers both patients and clinicians to understand and validate results. Open platforms that enable peer review and transparency can further strengthen credibility. Educating patients that AI tools complement—rather than replace—clinical judgment also eases concerns. Ultimately, shared decision-making that includes patients, physicians, and AI insights will lead to greater satisfaction and better care [144].

### 7.5. Barriers to AI-Auscultation Adoption

Despite these advantages, several barriers remain:

High-quality recordings can be difficult to obtain in busy clinical environments.

Most AI models are trained on small, homogenous datasets, increasing the risk of bias [145].

Clinician resistance due to reliance on traditional workflows and concerns about autonomy.

Lack of standardized protocols for integration into EHRs or clinical pathways.

Addressing these issues through training, validation studies, and inclusive design is essential for widespread adoption.

AI-enabled auscultation has the potential to bridge critical diagnostic delays in ILD care by offering an accessible, low-cost, real-time tool for early detection and monitoring. Rather than replacing existing diagnostics, it should be integrated into a multimodal diagnostic framework, enhancing accuracy, equity, and clinical decision-making across care settings.

## 8. Limitations

The review shows artificial intelligence demonstrates promising results for ILD diagnosis and management, yet the current literature reveals multiple repeating challenges that need resolution for clinical deployment. The restricted dataset dimensions of many research studies represent a main concern. The training and validation of most AI models occurred on patient cohorts that originated from one institution with limited numbers. The limited data range creates obstacles for general application since it does not cover diverse clinical groups with different imaging protocols and disease patterns. The dominance of data from a single institution creates institutional bias, which reduces the external applicability of findings. Studies that use a retrospective approach face major difficulties because of their research design. The selection bias and documentation inconsistencies, together with missing data quality standards, make retrospective data collection problematic. The reliability of research findings decreases because of these factors, which also prevent the evaluation of AI systems in real-time clinical practice. Most studies lacked external validation of their models through independent datasets, which remain essential to establish model robustness and prevent overfitting. Interpretability remains another important limitation. Deep learning-based models and their high performance do not provide clear insights into their decision-making processes. These models create a “black box” problem, meaning that the decision-making processes of these models are not easily understood by humans, making it difficult to explain why a particular diagnosis or decision was made. In healthcare, this lack of transparency can reduce clinician trust in the model’s predictions and hinder its integration into clinical workflows, which reduces clinician trust while making their implementation in clinical settings more difficult. The use of Grad-CAM and attention-based visualization techniques shows potential as interpretability solutions, yet their implementation remains irregular between research studies. Technical and infrastructural barriers also pose constraints. The deployment of several models faces challenges because they need high-performance computing resources together with complex preprocessing steps, which could make them unsuitable for regular or resource-limited settings. The different approaches to imaging protocols and labeling methods, and data annotation procedures between institutions create barriers to achieving reproducibility and comparative analysis. Most research studies concentrate on idiopathic pulmonary fibrosis (IPF) while ignoring other ILD subtypes and mixed-pathology cases. The current research demonstrates inadequate utilization of electronic health records along with laboratory data and genomic information, which could improve both model accuracy and clinical usefulness. The majority of research studies failed to evaluate clinical outcomes and workflow integration, so the effects of AI tools on patient care remain unknown. Real-world prospective evaluations and tests of diagnostic accuracy and time to diagnosis and treatment decisions have been performed only by a few models. The successful transition of ILD care AI tools to reliable clinical solutions requires multicenter collaborations and prospective validation, along with model explainability and multimodal data integration to overcome existing limitations.

As a narrative review, the synthesis presented here is inherently limited by heterogeneity across the included studies. Variability in auscultation devices, acoustic acquisition protocols, AI model architectures, labeling methods, and HRCT reference standards reduces the comparability of findings across studies. Many of the primary studies used small or single-center datasets, introducing potential selection bias and limiting generalizability. A majority of the ILD-AI literature relies on retrospective datasets, which may contain incomplete documentation, inconsistent diagnostic coding, and limited representation of early-stage disease. Additionally, publication bias may favor studies reporting positive model performance, while negative or null findings remain underreported. These limitations affect the strength of the conclusions and highlight the need for multicenter, prospective validation and standardized methodologies before AI-based auscultation can be reliably integrated into clinical pathways.

## 9. Conclusions

AI-enhanced auscultation offers a promising and accessible approach for the early detection and monitoring of interstitial lung disease (ILD). Current evidence indicates that AI models can identify subtle respiratory acoustic abnormalities linked to early fibrotic changes and may complement existing modalities such as HRCT and PFTs by supporting earlier identification of at-risk patients, particularly in primary care, telemedicine, and underserved settings. Its non-invasive, portable, and cost-effective nature positions AI-auscultation as a valuable adjunct within a multimodal diagnostic framework.

However, the available literature remains heterogeneous, and several barriers must be addressed before broad clinical implementation. Standardization of datasets, improvement of model generalizability, and rigorous clinician training are essential for reliable deployment. Ethical considerations—including data privacy, informed consent, equitable model performance, and clear attribution of legal responsibility—must also be prioritized. Enhancing model explainability and establishing regulatory pathways will be critical for clinician trust and safe integration into clinical workflows.

Future work should focus on real-time decision support capabilities, seamless interoperability with electronic medical records, and multicenter prospective validation. Integrating auscultatory data with imaging, biomarkers, and genomic information may enable a more comprehensive assessment of ILD. Ultimately, the clinical value of AI-auscultation tools will depend on responsible innovation, transparent governance, and robust evidence demonstrating accuracy, reliability, and real-world utility.

## Figures and Tables

**Table 1:** Characteristics of subtypes of ILD.

ILD Category	ILD Subtypes	Common Auscultation Findings	Prognosis
Idiopathic Interstitial Pneumonias (IIPs)	− Idiopathic Pulmonary Fibrosis (IPF) [20] − Acute Interstitial Pneumonia (AIP) [21] − Nonspecific Interstitial Pneumonia (NSIP) [22] − Desquamative Interstitial Pneumonia (DIP) [23] − Cryptogenic Organizing Pneumonia (COP) [24] − Respiratory Bronchiolitis-Associated ILD (RB-ILD) [25]	Fine velcro-like inspiratory crackles, typically basal	IPF: Poor (3–5 yr survival); Others: Good if treated early
Autoimmune and Connective Tissue Disease-Associated ILDs	− Lymphoid Interstitial Pneumonia (LIP) [26] − Rheumatoid Arthritis-Associated ILD (RA-ILD) [27] − Systemic Sclerosis-Associated ILD (SSc-ILD) [28] − Idiopathic Inflammatory Myopathy-Associated ILD (IIM-ILD) [29,30]	Fine bilateral basal crackles; systemic features vary	UIP pattern: Poor; NSIP or antibody-responsive types: Favorable
Exposure-Related ILDs	− Hypersensitivity Pneumonitis (HP) [31] − Asbestosis [32] − Silicosis [33] − Smoking-Related ILD [34] − Drug-Induced ILD (DI-ILD) [35] − Radiation-Induced ILD (RI-ILD) [36]	Fine inspiratory crackles (basal/mid/upper depending on etiology)	Prognosis varies: improves with early withdrawal of exposure; advanced fibrosis worsens outcome
Airspace-Filling ILDs	− Lymphangioleiomyomatosis (LAM) [37,38,39] − Pulmonary Langerhans Cell Histiocytosis (PLCH) [40,41,42,43] − Pulmonary Alveolar Proteinosis (PAP) [44]	Diffuse wheeze or fine crackles; pneumothorax in LAM/PLCH	LAM/PAP: Good prognosis with therapy; PLCH: Improves with smoking cessation
Granulomatous ILDs	− Sarcoidosis [45,46]	Fine crackles and wheeze (upper/mid zones); coarse crackles in advanced stages	Variable: Coarse crackles, B-symptoms, and advanced imaging findings indicate worse outcome
Other ILDs	− Acute Eosinophilic Pneumonia (AEP) [47] − Chronic Eosinophilic Pneumonia (CEP) [48,49] − Pulmonary Lymphangitic Carcinomatosis (PLC) [50,51]	Fine crackles (diffuse or lobe-specific); wheeze in CEP	AEP: Excellent recovery; CEP: Recurrences common; PLC: Very poor prognosis (~50% 2-month mortality)

**Table 2:** Comparing AI Models for Lung Sound-Based ILD Detection.

Model/Technique	Input Features	Feature Extraction	Application	Accuracy	Reference
CNN–LSTM	Raw audio + spectrogram	Spatial + temporal modeling	ILD progression monitoring	Not reported	[52]
Transformer (emerging)	MFCC/time-series	Self-attention across respiratory cycles	Breath cycle interpretation (experimental)	Not reported	[52]
DS-CNN	MFCC + STFT spectrograms	Time–frequency fusion	Velcro crackle detection	85.74%	[53]
Bi-ResNet	MFCC + STFT spectrograms	Bidirectional residual learning	Fine crackle classification	97.82%	[54]
Improved Random Forest	MFCC + db4 Wavelet	CFS + GR + SU + Ensemble decision trees	Multi-class respiratory sound classification	99.04%	[55]
AdaBoost	MFCC + db4 Wavelet	Ensemble boosting	Baseline ensemble for lung sound classification	96.63%	[55]
Gradient Boosting (GB)	MFCC + db4 Wavelet	Boosted decision trees	Alternate ensemble model	95.11%	[55]
Wavelet Transform (db4)	Raw lung sound	Time–frequency decomposition for transient events	Velcro crackle enhancement (preprocessing aid)	–	[52,55]

*p*-values and 95% confidence intervals are included where reported in the original studies. Several studies (e.g., Jung et al. [53], Wu et al. [54], and the improved random-forest ensemble [55]) did not provide CIs or *p*-values; therefore, only point estimates are presented.

**Table 3:** Summary of non-AI and AI-based diagnostic and prognostic modalities used in ILD, including techniques, findings, and limitations.

Non AI Traditional Methods:			
Year and Author	Study	Technique	Results and Limitations
2025, Boros et al. [60]	Value of Spirometry in Detecting Volume Restriction in ILD Patients	Retrospective, Cross-Sectional Analysis → Analyzed pulmonary function data from 1173 ILD patients over 5 years → Spirometry and whole-body plethysmography performed using MasterLab—‘Jaeger’ equipment following ERS standards → Compared TLC (Total Lung Capacity) and VC (Vital Capacity) for detecting volume restriction	Mean TLC was significantly lower than VC (93.7% vs. 98.0%, *p* < 0.001), with abnormal TLC more frequent (22.8%) than reduced VC (17.8%). VC showed 69.3% sensitivity and 88.5% PPV for restriction, but findings are limited to patients without airway obstruction, reducing applicability to more severe ILD.
2024, Kobayashi et al. [61]	ILD-NSCLC-GAP Scoring and Staging System	Modified ILD-GAP Index → A modified ILD-GAP index scoring system used to predict the incidence of ILD-AE and prognosis in patients with NSCLC and interstitial lung disease (ILD) → ILD subtypes, sex, age, and percent forced vital capacity (%FVC) are considered → The patients are categorized into stages I (0–1), II (2–3), and III (4–5) based on their index score → Data was collected from patients receiving platinum-based chemotherapy for NSCLC between 2002 and 2014	The modified ILD-GAP index predicted ILD-AE incidence and prognosis in NSCLC with ILD, showing higher risk and lower one-year survival with increasing scores (70.8% in stage I vs. 10% in stage III). Findings are limited by retrospective, single-center data and exclusion of patients receiving some chemotherapy or radiotherapy regimens.
2024, Hirata et al. [62]	ILD-GAPM Model for Prognosis in ILD	Retrospective, Observational Study → Analyzed 179 ILD patients (including IPF, iNSIP, CVD-IP, CHP, and UC-ILD) → Compared the ILD-GAP scoring system and ILD-GAPM, which combines ILD-GAP with the monocyte ratio → Pulmonary function tests (PFTs), HRCT, and clinical parameters were used to assess disease progression and prognosis	ILD-GAPM outperformed ILD-GAP in predicting 3-year ILD-related events (AUC: 0.747 vs. 0.710). Significant differences were observed in Kaplan–Meier survival curves based on monocyte ratio. ILD-GAPM was especially effective for predicting IPF progression. However, limitations include the single-center design, small sample size, and lack of longitudinal data for monocyte ratio changes.
2024, Thrainsson et al. [63]	VATS for Diagnosing ILD	Surgical Lung Biopsy (SLB) with VATS → Retrospective cohort study involving 68 patients who underwent VATS lung biopsy for suspected ILD → Preoperative tests included CT scans, spirometry, and bronchoscopy	VATS achieved a 92.6% diagnostic yield, most often diagnosing NSIP (29.4%) and UIP (23.5%). Limitations include the retrospective nature, small sample size, and lack of AI integration in the diagnostic process.
2023, Zanatta et al. [64]	CCL18 as a Biomarker for ILD and PF-ILD in Idiopathic Inflammatory Myopathies	Prospective Cohort Study → Analyzed serum levels of CCL18 and OX40L in 93 IIMs patients → HRCT was used to detect ILD and classify it into patterns (NSIP, UIP, OP) → PFTs were measured to evaluate lung function at baseline and at 24 months	CCL18 serum levels were significantly higher in IIMs-ILD patients (*p* < 0.0001). High CCL18 levels were independently associated with PF-ILD at the 24-month follow-up. The study found CCL18 to be a reliable predictor for PF-ILD (OR 1.006, *p* = 0.005). The ROC curve for CCL18 identified an optimal threshold of 303.5 ng/mL, with 82% sensitivity and 83% NPV. Limitations include the small sample size and lack of external validation.
2022, Le Guen et al. [65]	Clinical Impact of Surgical Lung Biopsy for Interstitial Lung Disease in a Reference Center	Patient Selection → Elective surgical lung biopsy for suspected ILD → Preoperative Multidisciplinary Discussion (MDD) (to confirm indication and biopsy sites) → Pre-op evaluation → Surgical Lung Biopsy (VATS or open) → Post-op care & monitoring → MDD1 (without biopsy results) → MDD2 (with biopsy results) → Compare MDD1 vs. MDD2 → Assess diagnostic & treatment changes → Record complications & outcomes (90-day follow-up) → Statistical analysis	In this study of 73 ILD patients, surgical lung biopsy (SLB) was safe, with no deaths and a 17% complication rate. It provided a definitive diagnosis in 95% of cases and led to changes in diagnosis and treatment in 48% and 45% of patients, respectively. SLB remains a valuable tool when noninvasive methods are inconclusive. The study’s main limitations include its retrospective, single-center design, which may affect generalizability.
2022, McDermott G et al. [66]	Associations of the MUC5B promoter variant with timing of interstitial lung disease and rheumatoid arthritis onset	Cohort identification and recruitment → DNA extraction → Genotyping of MUC5B promoter variant (rs35705950) → Collection of clinical data (ILD and RA onset) → Integration of genetic and clinical datasets → Statistical analysis (survival and regression models) → Clinical interpretation of association with disease progression.	The MUC5B promoter variant was associated with earlier onset of ILD in rheumatoid arthritis (RA) patients but did not influence timing of RA onset. Carriers developed ILD sooner after RA diagnosis, underscoring its role in early lung disease progression and risk prediction in RA.
2022, Gui et al. [67]	Role of Anti-Ro52 Antibodies in IIM-ILD Prognosis	Serological and Clinical Data Analysis → Retrospective cohort study of 267 IIM-ILD patients with various myositis-specific autoantibodies (MSAs) → Anti-Ro52 antibodies were detected in anti-MDA5 and anti-Jo1 positive patients using immunoblot assays → Clinical, laboratory, and imaging data were analyzed to assess the association between anti-Ro52 positivity and disease progression	Anti-Ro52 antibody positivity was linked to a higher risk of rapidly progressive ILD, worse prognosis, and greater mortality, particularly in patients also carrying anti-MDA5 antibodies, who showed more severe clinical features including Gottron sign. Generalizability is limited by the retrospective design and absence of longitudinal follow-up.
2022, Oishi et al. [68]	1-Minute Sit-to-Stand Test for Desaturation in ILD	Retrospective Observational Study → Compared 1 min sit-to-stand test (1STST) with the 6 min walk test (6MWT) in 116 ILD patients → Measured pulse oxygen saturation (SpO_2_) nadir during both tests → Analyzed correlation and agreement between 1STST and 6MWT for desaturation detection	Nadir SpO_2_ during the 1STST strongly correlated with the 6MWT (ρ = 0.82, *p* < 0.0001) and showed high agreement (κ = 0.82) in detecting desaturation <90%. The 1STST outperformed DLCO for identifying desaturation, though findings are limited by the retrospective design and possible selection bias.
2022, Harari et al. [69]	The 6-Minute Walk Test (6MWT) as a Primary End-Point in ILD	Literature Review & Methodological Analysis → Reviewed multiple ILD trials and studies using 6MWT as a primary endpoint in clinical trials → Focused on 6MWD (6 min walk distance) and oxygen desaturation	The 6MWD emerged as a reliable endpoint for ILD trials, particularly in advanced ILD and ILD-PH, where it predicted clinical worsening and disease progression. Interpretation is limited by variability in 6MWT methodology, influence of patient factors and environment, and a “floor effect” in advanced disease that reduces sensitivity to change over time.
2021, Yan et al. [70]	Lung Ultrasound (LUS) for ILD Detection	Lung Ultrasound (LUS) → A semi-quantitative approach using B-lines to assess ILD severity → 14 intercostal spaces (ICSs) were assessed using a GE-E9 Doppler ultrasound machine with a line array probe → B-line scores were calculated based on the number of B-lines observed, with scoring for mild (6–15 B-lines), moderate (16–30 B-lines), and severe (>30 B-lines) cases	LUS showed 93% sensitivity and 73% specificity for ILD detection, with 94% PPV and 67% NPV. LUS was found to be an effective, non-invasive, radiation-free screening tool for ILD, though it performed slightly worse than HRCT, with some false negatives in early-stage ILD. The study was limited by its retrospective design and small sample size, as well as challenges in detecting deep lung abnormalities.
2021, Matson et al. [71]	MMP-7 as a Prognostic Biomarker in Scleroderma-Associated ILD	Retrospective Cohort Study → Analyzed serum levels of MMP-7, CXCL13, and CCL18 in 115 SSc-ILD patients → HRCT, PFTs, and lung biopsy were used to assess disease severity and lung function → Immunohistochemistry and qPCR were used to measure MMP-7 expression in lung tissue	Higher MMP-7 levels were significantly associated with lower FVC and DLCO (*p* < 0.001), indicating worse lung function. Patients with higher MMP-7 had an increased risk of death or lung transplant (HR = 2.05, *p* = 0.009). MMP-7 levels categorized into low, medium, and high tertiles correlated with disease severity (*p* < 0.001). Limitations include the lack of a validation cohort and the study being limited to a single-center cohort.
2020, Kim et al. [72]	KL-6 as a Prognostic Biomarker in RA-ILD	KL-6 Assay → Measured KL-6 levels in plasma using the Nanopia KL-6 assay (latex-enhanced immunoturbidimetric method) → 84 RA-ILD patients included in the study, with HRCT scans used to identify UIP patterns → Multivariate logistic regression and Cox proportional hazard analyses were used to assess the relationship between KL-6 levels and disease prognosis	High KL-6 levels (>685 U/mL) were found to be independently associated with increased mortality risk (HR = 2.984, *p* = 0.016) and UIP patterns (OR = 5.173, *p* = 0.005). The study found that KL-6 could serve as a reliable prognostic biomarker for RA-ILD, especially in those with a UIP pattern, suggesting its potential role in early disease assessment. However, limitations included the small sample size and lack of longitudinal data to further confirm findings.
2020, Pugashetti et al. [73]	ILD-Screen: A Diagnostic Prediction Tool for ILD	Prospective Cohort Study → Developed ILD-Screen, a diagnostic tool derived from PFT variables like TLC, FEV1, DLCO, and clinical factors → Logistic regression models identified predictive factors for ILD → Validated in independent cohorts and applied prospectively over a 1-year period	ILD-Screen showed 79% sensitivity and 83% specificity in identifying ILD cases. It improved diagnostic workflow by increasing the rate of chest CT imaging and reducing time to diagnosis. Prospective validation showed that ILD-Screen significantly outperformed clinical features like dyspnea and cough. However, limitations include selection bias and the need for further validation in broader patient populations.
2019, Newton et al. [74]	Telomere length and genetic variant associations with interstitial lung disease progression and survival	Patient Enrollment & Sample Collection → Clinical Data Collection (Demographics, ILD subtype, progression, survival) → DNA Extraction from Blood Samples → Measurement of Telomere Length (qPCR or Southern blot assay) → Genotyping of Genetic Variants (SNP arrays or sequencing) → Statistical Analysis (Associations of telomere length & genetic variants with ILD progression and survival) → Interpretation of Results & Correlation with Clinical Outcomes	Shorter telomere length in ILD was linked to faster disease progression and reduced survival, with genetic variants in telomere maintenance also influencing outcomes. These findings support telomere biology as a prognostic marker and potential therapeutic target. Interpretation is limited by the observational design, small sample size for rare variants, use of a specialized cohort, and reliance on blood-cell telomere length, which may not reflect lung tissue status.
2017, Yamakawa et al. [75]	KL-6 and SP-D as Predictive Biomarkers for ILD in SSc/MCTD	Retrospective Cohort Study → Analyzed serum KL-6 and SP-D levels in 40 patients (29 with SSc and 11 with MCTD) → Pulmonary function tests (FVC, DLCO) and HRCT were used to evaluate ILD severity and progression	KL-6 levels correlated with DLCO and HRCT disease extent, while changes in KL-6 were associated with FVC decline. SP-D was identified as a significant predictor of FVC decline. The study suggested KL-6 for monitoring and SP-D for predicting FVC decline. Limitations include the small sample size and the retrospective nature of the study.
2017, Kropski et al. [76]	Genetic Testing in Idiopathic Pulmonary Fibrosis (IPF)	Literature Review & Cohort Study → This study reviewed genetic contributions to familial interstitial pneumonia (FIP) and idiopathic pulmonary fibrosis (IPF), focusing on genetic variants like those related to telomerase and surfactant proteins → The study also examined the role of genetic testing for families at high risk of IPF, providing insights into screening and counseling protocols	Mutations in TERT, SFTPC, and SFTPA2 were found to significantly contribute to familial IPF cases. The study suggests genetic testing for at-risk families to detect telomere dysfunction. However, the study acknowledged limitations such as limited data on disease penetrance and the lack of standardized clinical guidelines for genetic screening in routine practice.
2015, Bauer et al. [77]	Influence of Autoimmune Biomarkers on ILD Prognosis	Case-Control Study → Retrospective study on 3573 ILD patients at Mayo Clinic, Rochester → Assessed autoimmune biomarkers like ANA, RF, and aldolase through serological tests → Analyzed associations with ILD, adjusting for age, gender, race, smoking history, and CTD	Positive ANA and RF were significantly associated with increased odds of ILD. ANA remained an independent risk factor for ILD after adjustments (OR 1.70, 95% CI 1.33–2.17). Patients with ILD alone had poorer survival than those with CTD-ILD (*p* = 0.001), and positive biomarkers did not improve prognosis. The study’s retrospective design and biases in biomarker testing due to the varied application of tests across the patient population were key limitations.
**AI-Based Methods:**			
**Year and Author**	**Study**	**Technique**	**Results and Limitations**
2025, Guo et al. [78]	AI and Machine Learning for IPF Diagnostics	Neural Network Model → Utilized gene expression datasets from the GEO database to identify differentially expressed genes (DEGs) in IPF vs. healthy controls → Employed Lasso regression and Random Forest algorithms to screen for potential biomarkers → Artificial Neural Network (ANN) built for IPF classification, with ROC curve and AUC metrics for diagnostic performance → Immune infiltration analysis conducted with the CIBERSORT tool to explore immune cell interactions	The neural network model showed excellent diagnostic performance, with AUC values for biomarkers ASPN, COMP, and GPX8 being 0.94, 0.99, and 0.94, respectively. The immune analysis revealed significant changes in immune cell populations in IPF, highlighting their role in pathogenesis. Limitations include small sample sizes and the inherent “black-box” nature of the ANN model, which may affect interpretability.
2025, Gompelmann et al. [79]	AI-powered evaluation of lung function for diagnosis of interstitial lung disease.	Data Collection → Data Preprocessing → Feature Extraction → AI Model Training → AI Evaluation → Model Validation → Result Interpretation → Diagnosis of ILD.	AI support improved pulmonologists’ interpretation of pulmonary function tests, enhancing diagnostic accuracy and enabling earlier ILD detection. Findings are limited by the small, retrospective dataset, short AI exposure (4–6 months), and lack of evaluation of long-term outcomes or comparisons across different AI models.
2025, Guiot et al. [80]	Automated AI-based image analysis for quantification and prediction of interstitial lung disease in systemic sclerosis patients	Patient selection & CT acquisition → Image preprocessing (normalization, noise reduction) → AI model training on annotated CT scans (ML/DL algorithms) → Automated lung region segmentation → Quantification of ILD features (fibrosis, ground-glass opacity) → Extraction of imaging biomarkers → Statistical analysis & validation against clinical/functional data → Prediction of ILD progression and outcomes.	AI-based image analysis accurately quantified ILD in systemic sclerosis, correlating well with clinical tests and predicting progression more effectively than traditional methods, offering faster and more consistent assessment. Its broader use is limited by the single-disease focus, retrospective design, and absence of long-term prospective validation.
2024, Teramachi et al. [57]	Deep Learning for Predicting Acute Exacerbation and Mortality in ILD	Long Short-Term Memory (LSTM) network → Applied to longitudinal ILD patient data for predicting acute exacerbations (AE-ILD) and mortality → Managed temporal gaps through data imputation → Incorporated key features such as neutrophil counts, CRP, ILD-GAP score, and environmental exposures (e.g., particulate matter).	The LSTM model demonstrated higher prediction accuracy than Cox Proportional Hazards models, with C-index values of 0.78–0.85 in internal and external validation cohorts. Its use is limited by retrospective data, missing external predictors such as viral infections, and challenges in incorporating environmental variables effectively.
2024, Huang et al. [81]	Proteomics-Based Classifier for ILD Diagnosis	Machine learning on plasma proteomics → High-throughput assays used to analyze plasma from 1247 IPF and 352 CTD-ILD patients → Recursive Feature Elimination (RFE) for feature selection → Four models (Support Vector Machine, LASSO regression, Random Forest, Imbalanced Random Forest) trained on selected features for diagnosis.	The proteomic classifier (PC37) achieved strong diagnostic performance with AUCs of 0.85–0.90 in test cohorts and 0.94–0.96 in external datasets, with sensitivity of 78.6–80.4% and specificity of 76–84.4%. The composite diagnosis score model reached 82.9% accuracy for single-sample classification. Generalizability is limited by reliance on blood-based biomarkers and the relatively small training and testing datasets.
2024, Felder et al. [82]	Computer-Based Imaging in ILD: QCT and AI	Quantitative Computed Tomography (QCT) and AI-based image analysis → Utilized deep learning algorithms (e.g., SOFIA, DTA) for pattern identification and progression prediction on HRCT images → Textural analysis and data-driven techniques were applied to quantify fibrosis, honeycombing, and ground-glass opacities (GGO)	QCT with AI provided more reliable and precise measurements than traditional semiquantitative methods, improving disease progression predictions and facilitating early detection of fibrotic changes. However, challenges include data privacy concerns, integration with clinical practice, and the need for explainable AI models to increase clinician trust.
2024, Guerra et al. [83]	U-Net CNN for CT Scan Analysis in Progressive Fibrotic ILD	U-Net Convolutional Neural Network (CNN) → Developed U-Net CNN for quantification of ILD progression in CT scans → 32 patients with fibrotic ILD (IPF, i-NSIP, u-IIP) were included → CT scans were processed to identify fibrotic changes and correlate these changes with FVC (Forced Vital Capacity)	The U-Net CNN showed a significant correlation between ILD% and FVC decline (r = –0.30, *p* = 0.004). An ILD progression rate ≥4%/year was linked to poor prognosis (*p* = 0.001), with ROC analysis yielding an AUC of 0.83, indicating good predictive accuracy. Limitations include the small sample size and the need for external validation.
2024, Xu et al. [84]	Classification and Recognition of Lung Sounds Using Artificial Intelligence and Machine Learning: A Literature Review.	Record lung sounds → Remove noise from the recordings → Extract patterns or frequency → Enhance the Data → Choose and Train a machine leaning Model → Classify the Sounds → Evaluate Performance.	AI and machine learning methods can accurately classify lung sounds, with deep learning models outperforming traditional approaches. Model performance improves with careful feature selection and incorporation of additional datasets, supporting faster and more accurate respiratory diagnoses. Broader clinical use requires further validation to ensure reliability and ease of application in real-world settings.
2024, Román Ivorra JA et al. [85]	Prevalence and clinical characteristics of patients with rheumatoid arthritis with interstitial lung disease using unstructured healthcare data and machine learning	Identification of Rheumatoid Arthritis Cohort → Extraction of Unstructured Clinical Notes → Natural Language Processing (NLP) Preprocessing → Creation of ILD-related indicators (fibrosis, honeycombing, ground glass) → Machine Learning Model Development (e.g., random forest) → Assess performance (AUC, sensitivity, specificity) → ILD Prevalence Estimation & Clinical Correlation	A machine-learning model detected ILD in 6.9% of more than 22,000 RA patients—higher than rates identified with structured data alone. Using NLP with machine learning on free-text notes revealed clinically relevant patterns and improved early ILD detection in RA. Findings are limited by the retrospective design, dependence on unstructured notes with variable quality, and uncertain generalizability across healthcare systems without external validation.
2023, Rea et al. [86]	AI and Quantitative Imaging in ILD Diagnosis	AI-based image analysis → Integration with HRCT to detect ILD patterns → Quantitative software tools provide accurate, repeatable, and objective lung assessments → AI supports recognition and classification of features such as ground-glass opacities, honeycombing, and fibrosis → Models also incorporate clinical, genomic, and proteomic metadata to enhance diagnostic accuracy.	AI-enhanced HRCT analysis improved diagnostic precision compared with visual interpretation, providing quantitative, reproducible assessments and reducing observer bias. Its broader use is limited by variable performance across populations, the need for large training datasets, and incomplete integration of clinical variables such as biomarkers.
2023, Dianat et al. [87]	Classification of Pulmonary Sounds for ILD Diagnosis	Deep Learning-based Classification → Used Convolutional Neural Networks (CNNs) for classifying pulmonary sounds recorded with a digital stethoscope → Pre-processing pipeline involved Variational Mode Decomposition (VMD) for noise reduction and data augmentation using techniques like SpecAugment → Focused on identifying velcro crackles associated with ILD, particularly in patients with connective tissue diseases (CTD)	The model achieved 91–93% diagnostic accuracy in classifying lung sounds, showing strong potential for use in large-scale screening programs. The pre-processing with VMD and SpecAugment significantly improved model performance. However, limitations include potential sensitivity to background noise in real-world applications and the need for high-quality auscultation data. The model’s ability to generalize to other forms of ILD requires further validation across diverse clinical settings.
2023, Das et al. [88]	Collaboration between Pulmonologists and explainable AI (XAI)	Multicenter, Intervention Study → Pulmonologists interpreted 24 PFT reports in two steps: control (without XAI suggestions) and intervention (with XAI suggestions) → The study involved two phases: monocentric (P1) with 16 pulmonologists and multicentric (P2) with 62 pulmonologists → XAI suggestions were generated using a machine learning model predicting diseases like COPD, ILD, and asthma with Shapley values (SVs) providing transparency into AI decisions	With XAI support, diagnostic accuracy improved significantly—preferential by 10.4% and differential by 9.4% in Phase 1, and by 5.4% and 8.7% in Phase 2 (all *p* < 0.001). Pulmonologists still outperformed the model, but results highlight XAI as a useful adjunct in diagnostic decision-making. The effect may have been reduced by deliberately including incorrect AI suggestions, while limitations include small sample size and possible learning effects from repeated testing.
2023, Beverin et al. [58]	Predicting TLC from Spirometry Data Using Machine Learning	Machine Learning Model Development → Three tree-based models (CatBoost, XGBoost, and Random Forest) were trained on 51,761 spirometry data points to predict total lung capacity (TLC) → The models used patient characteristics (age, height, gender, weight) and spirometry metrics (FVC, FEV1, etc.) → The best model (CatBoost) was validated on an independent test set of 1402 patients	The CatBoost model predicted TLC with a mean squared error (MSE) of 560.1 mL. It showed high sensitivity (83%) and specificity (92%) in identifying restrictive ventilatory impairment. Limitations include the study’s reliance on Caucasian patient data, the potential overestimation of TLC in obstructive diseases, and the absence of validation in non-Caucasian populations.
2023, Wu et al. [56]	Identifying potential biomarkers of idiopathic pulmonary fibrosis through machine learning analysis	Gene expression data collection (IPF and controls) → Preprocessing (quality control, normalization, filtering) → Identification of differentially expressed genes → Feature selection with machine learning (Random Forest, SVM, LASSO) → Model training and validation for IPF classification → Biomarker selection based on feature importance → Functional enrichment and pathway analysis → Verification through literature and experimental evidence.	Machine learning on gene expression data identified key biomarkers for IPF through differential gene analysis and feature selection, with further biological analysis linking them to fibrosis and immune pathways. These biomarkers show potential for early diagnosis and treatment guidance. Generalizability is limited by the small, non-diverse public datasets, reliance on retrospective validation, and lack of functional or multi-omics integration such as proteomics.
2023, Pan et al. [89]	Unsupervised machine learning identifies predictive progression markers of IPF	Data collection (HRCT scans of IPF patients) → Preprocessing (normalization, segmentation) → Feature extraction (radiomic/imaging features) → Dimensionality reduction (e.g., PCA) → Unsupervised clustering (k-means, hierarchical) → Identification of patient subgroups → Analysis of progression markers by comparing clinical outcomes and imaging features across clusters → Validation of predictive markers.	Unsupervised machine learning on CT scans identified distinct IPF patient subgroups with different progression patterns, with specific imaging features predicting faster worsening and aiding risk stratification. Generalizability is limited by the small, retrospective, single-center datasets and the exclusion of clinical or molecular variables, highlighting the need for larger prospective studies.
2023, Exarchos KP et al. [90]	Recent Advances of Artificial Intelligence Applications in Interstitial Lung Diseases	Data collection → Preprocessing of medical data (CT scans, clinical parameters, lung function tests) → Feature extraction (radiomic features, biomarkers, PFT indices) → AI model development (ML/DL methods: CNN, SVM, etc.) → Model training and validation with annotated datasets → Evaluation (accuracy, sensitivity, specificity, ROC) → Clinical application for diagnosis, prognosis, and disease monitoring → Integration into workflow as decision-support and radiology assistance tools.	AI, particularly CNN-based deep learning on HRCT, achieved performance comparable to expert radiologists in classifying ILD subtypes, assessing severity, and predicting progression. Incorporating clinical and functional data further improved diagnostic accuracy and patient stratification, supporting AI-driven decision tools. Limitations include reliance on small, heterogeneous datasets, lack of standardized imaging and annotation, limited interpretability of “black-box” models, and scarce prospective validation or clinical integration.
2022, Wu X et al. [91]	Idiopathic Pulmonary Fibrosis Mortality Risk Prediction Based on Artificial Intelligence: The CTPF Model	Gather clinical data and HRCT images of IPF patients → Extract quantitative CT features and clinical parameters → Data Preprocessing → Apply techniques like LASSO to identify key predictors → Train machine learning models → Assess model performance using AUC, sensitivity, and specificity → Select the best model and integrate into the CTPF system → Predict individual patient mortality risk	The CTPF model, combining clinical and CT imaging features, predicted mortality risk in IPF patients, with logistic regression achieving the best performance (AUC 0.85) for stratifying patients into high- and low-risk groups. Limitations include the retrospective, single-center design, small sample size, reliance on specific CT software, and lack of external validation, reducing generalizability until confirmed in broader cohorts.
2022, Pawar et al. [92]	Two-Stage Hybrid Approach of Deep Learning Networks for Interstitial Lung Disease Classification	Data Acquisition → Preprocessing (e.g., resizing, normalization, augmentation) → Stage 1—Feature Extraction using CNN Model 1 → Stage 2—Feature Extraction using CNN Model 2 → Feature Fusion (combining features from both CNNs) → Classification Layer (fully connected layers + softmax) → Output: ILD Class Prediction	Pawar and Talbar’s two-stage hybrid deep learning method, combining features from two CNN models, improved ILD image classification accuracy compared with single models and traditional approaches, offering a promising tool to support radiologists. Its broader application is limited by the small dataset, high computational demands, lack of clinical data integration, and absence of extensive independent validation.
2021, Hwang et al. [93]	CBIR System for DILD Diagnosis Using CT	Content-Based Image Retrieval (CBIR) → Lung segmentation using deep CNNs → Classification of six DILD patterns (honeycombing, reticular opacity, emphysema, ground-glass opacity, consolidation, normal lung) → Quantification of these patterns across HRCT slices → Feature extraction and similarity assessment using Euclidean distance to match query CTs with database images.	The CBIR system achieved 61.7% top-1 retrieval accuracy and 81.7% within the top-5, performing best for UIP, where 96.7% of retrieved CTs matched the query class. It showed promise in supporting radiologists, but performance varied across ILD patterns (notably lower in COP) and requires validation on larger, more diverse datasets.
2021, Wong et al. [94]	Fibrosis-Net: A Tailored Deep Convolutional Neural Network Design for Prediction of Pulmonary Fibrosis Progression From Chest CT Images	Preprocessing of Images by CT → CT scans divided into 2D axial image patches → Each patch labeled based on fibrosis progression (stable vs. progressive) → Custom Deep Convolutional Neural Network (CNN) architecture → Training the CNN → Model performance evaluated on validation/test sets → Use of Grad-CAM (Gradient-weighted Class Activation Mapping) for visualizing regions contributing to prediction → Prediction of Pulmonary Fibrosis Progression.	The study demonstrated that Fibrosis-Net, a customized deep learning model, can be used as a non-invasive, image-based tool to predict pulmonary fibrosis progression, potentially aiding clinicians in early risk stratification and treatment decisions. The study used a small, single-center dataset and only CT images, limiting generalizability. It offered binary predictions without clinical data and had limited interpretability despite Grad-CAM. Being retrospective, it may also include bias.
2021, Zhang et al. [95]	Real-World Verification of Artificial Intelligence Algorithm-Assisted Auscultation of Breath Sounds in Children.	Collect breath sounds from pediatric patients → Preprocess and clean audio recordings → Input processed audio into the AI algorithm → Extract relevant acoustic features from breath sounds → Classify breath sounds into categories (e.g., normal, wheeze, crackle) → Generate AI-assisted diagnostic results → Compare AI outputs with clinical evaluation for validation.	An AI-assisted auscultation system accurately detected abnormal breath sounds such as wheezes and crackles in children, demonstrating reliability in real-world clinical settings and supporting more consistent diagnoses where expert auscultation is limited. Its generalizability is restricted by the small, single-center cohort, potential susceptibility to background noise and recording variability, reliance on subjective clinician comparisons, and the absence of multicenter validation.
2020, Romei et al. [96]	Automated CT Analysis for IPF Severity and Progression	CALIPER software → Automated lung parenchyma segmentation → Classification into CT patterns (reticular, honeycombing, ground-glass, normal) → Quantification of fibrosis and PVRS as % lung volume → Provides reproducible HRCT assessment of disease progression without manual input.	The study demonstrated that CALIPER-derived parameters, specifically ILD% and PVRS%, were strongly correlated with Forced Vital Capacity (FVC) measurements, offering a reliable tool for tracking disease progression. However, the study had limitations due to its retrospective nature and sample size, requiring further validation with larger, prospective studies.
2019, Pang et al. [97]	Automatic Lung Segmentation for ILD Diagnosis	Hybrid Segmentation Model → Combines texture features and deep features for lung segmentation → Texture features are extracted using Gray-Level Co-occurrence Matrix (GLCM) to analyze lung patterns → Deep features are obtained using U-Net, a convolutional neural network (CNN), to refine segmentation → Images are pre-processed using Wiener filtering to remove noise from HRCT images	The segmentation model achieved high accuracy with a Dice Similarity Coefficient of 89.4%, comparable to state-of-the-art methods. By combining texture and deep learning features, segmentation performance was enhanced. Limitations include reliance on well-annotated datasets and sensitivity to noise or image quality, which may hinder real-world application
2017, Jacob et al. [98]	Using Computer-Based CT Analysis for Mortality Prediction in Idiopathic Pulmonary Fibrosis	CALIPER tool → Lung tissue segmentation using density-based morphology → Airway segmentation via 3D region-growing algorithm → Pulmonary vessel detection with multi-scale tubular enhancement filters → Vessel volume quantification by size thresholds (PVV < 5 mm^2^, PVV < 10 mm^2^, PVV > 5 mm^2^) → Extraction of ILD features (ground-glass opacities, honeycombing, reticular patterns) using texture analysis and image processing.	CALIPER-derived measurements, particularly pulmonary vessel volume (PVV), were stronger predictors of mortality than visual CT scores. PVV, honeycombing, and CPI combined with CALIPER data emerged as key mortality indicators. Limitations include the lack of external validation and the need for further refinement of segmentation algorithms to improve predictive accuracy.
2016, Shin et al. [99]	Deep Convolutional Neural Networks for Computer-Aided Detection	CNN architectures (CifarNet, AlexNet, GoogLeNet) → Detection of thoraco-abdominal lymph nodes and ILD classification → Training with random 2.5D CT views → Transfer learning from ImageNet pre-trained models → Fine-tuning for medical imaging applications.	The model achieved high sensitivity (86%) for mediastinal lymph node detection while reducing false positives to three per patient. Transfer learning improved ILD classification accuracy, but the small dataset (120 patients) limits generalizability.

## Data Availability

This review was based on publicly available academic literature databases.
